# Advances in methylation analysis of liquid biopsy in early cancer detection of colorectal and lung cancer

**DOI:** 10.1038/s41598-023-40611-w

**Published:** 2023-08-19

**Authors:** Hyuk-Jung Kwon, Sun Hye Shin, Hyun Ho Kim, Na Young Min, YuGyeong Lim, Tae-woon Joo, Kyoung Joo Lee, Min-Seon Jeong, Hyojung Kim, Seon-young Yun, YoonHee Kim, Dabin Park, Joungsu Joo, Jin-Sik Bae, Sunghoon Lee, Byeong-Ho Jeong, Kyungjong Lee, Hayemin Lee, Hong Kwan Kim, Kyongchol Kim, Sang-Won Um, Changhyeok An, Min Seob Lee

**Affiliations:** 1 R&D Department, Eone-Diagnomics Genome Center, Inc., 143 Gaetbeol-Ro, Yeonsu-Gu, Incheon, 21999 Republic of Korea; 2 Division of Pulmonary and Critical Care Medicine, Department of Medicine, Samsung Medical Center, Sungkyunkwan University School of Medicine, 81 Irwon-Ro, Gangnam-Gu, Seoul, 06351 Republic of Korea; 3 Department of Surgery, Bucheon St. Mary’s Hospital, College of Medicine, The Catholic University of Korea, 327 Sosa-Ro, Bucheon, 14647 Republic of Korea; 4 Department of Thoracic and Cardiovascular Surgery, Samsung Medical Center, Sungkyunkwan University School of Medicine, 81 Irwon-Ro, Gangnam-Gu, Seoul, 06351 Republic of Korea; 5 Gangnam Major Hospital, 452 Dogok-Ro, Gangnam-Gu, Seoul, 06279 Republic of Korea; 6 Diagnomics, Inc., 5795 Kearny Villa Rd., San Diego, CA 92123 USA

**Keywords:** Cancer genomics, Lung cancer, Colorectal cancer, Cancer screening

## Abstract

Methylation patterns in cell-free DNA (cfDNA) have emerged as a promising genomic feature for detecting the presence of cancer and determining its origin. The purpose of this study was to evaluate the diagnostic performance of methylation-sensitive restriction enzyme digestion followed by sequencing (MRE-Seq) using cfDNA, and to investigate the cancer signal origin (CSO) of the cancer using a deep neural network (DNN) analyses for liquid biopsy of colorectal and lung cancer. We developed a selective MRE-Seq method with DNN learning-based prediction model using demethylated-sequence-depth patterns from 63,266 CpG sites using *Sac*II enzyme digestion. A total of 191 patients with stage I–IV cancers (95 lung cancers and 96 colorectal cancers) and 126 noncancer participants were enrolled in this study. Our study showed an area under the receiver operating characteristic curve (AUC) of 0.978 with a sensitivity of 78.1% for colorectal cancer, and an AUC of 0.956 with a sensitivity of 66.3% for lung cancer, both at a specificity of 99.2%. For colorectal cancer, sensitivities for stages I–IV ranged from 76.2 to 83.3% while for lung cancer, sensitivities for stages I–IV ranged from 44.4 to 78.9%, both again at a specificity of 99.2%. The CSO model's true-positive rates were 94.4% and 89.9% for colorectal and lung cancers, respectively. The MRE-Seq was found to be a useful method for detecting global hypomethylation patterns in liquid biopsy samples and accurately diagnosing colorectal and lung cancers, as well as determining CSO of the cancer using DNN analysis.

Trial registration: This trial was registered at ClinicalTrials.gov (registration number: NCT 04253509) for lung cancer on 5 February 2020, https://clinicaltrials.gov/ct2/show/NCT04253509. Colorectal cancer samples were retrospectively registered at CRIS (Clinical Research Information Service, registration number: KCT0008037) on 23 December 2022, https://cris.nih.go.kr, https://who.init/ictrp. Healthy control samples were retrospectively registered.

## Introduction

Cancer is a major public health concern, with an estimated 19.3 million new cancer cases and 10 million cancer deaths globally in 2020. Early detection of cancer is crucial for improving survival rates and reducing the burden of the disease. Liquid biopsy, a non-invasive diagnostic method that detects circulating tumor cells or cancer-derived nucleic acids in blood or urine, has shown promise for early cancer detection and diagnosis. Tissue biopsy is the gold standard for cancer diagnosis, but it is invasive, expensive, and may not always be feasible. Tissue biopsy involves the removal of a small piece of tissue from the suspected cancer site, typically through a surgical procedure. This method can only provide information about the specific area where the sample was taken. In contrast, liquid biopsy is a less invasive method that involves analyzing blood or other bodily fluids for signs of cancer cells or genetic material^1,2^. Cell-free DNA (cfDNA) liquid biopsy has been extensively studied for its potential in detecting cancer signals at an early stage and predicting the course of the disease^3–5^. Cancer genome analysis using next-generation sequencing (NGS) technologies involves sequencing and analyzing the DNA of cancer cells to identify genetic mutations known as hot-spot regions (such as KRAS and PIK3CA) that are associated with the development and progression of cancer. There are several different approaches to cancer genome analysis using NGS such as whole genome sequencing (WGS), whole exome sequencing (WES) and targeted sequencing^6,7^. Hot spot mutation analysis using target sequencing panel, which focuses on detecting specific mutations that are commonly found in cancer, can be limited in its ability to detect cancer at an early stage for the following reasons. First, some cancer cells may not have mutations to be detected by hot spot analysis. In cases of lung adenocarcinoma (LUAD), a common hot-spot mutation is absent in about 20% of patients^8^. Second, most hot spot mutation is rare even when it is detected in patients. This can be a limitation in the detection of circulating tumor DNA (ctDNA) in blood samples, where a small amount of ctDNA is often mixed with a large amount of normal cfDNA. In such cases, relying on the detection of a single mutation may not be sensitive enough, leading to a higher rate of false negatives (FNR)^9^. To overcome these limitations, researchers are exploring several alternative approaches. One such approach involves analyzing the fragmentation patterns of cfDNA, such as read length, end-motif sequences, and chromosomal distribution, using low-coverage whole genome sequencing analysis. This method has shown promising results in diagnosing certain cancer compared to mutation-based analysis and may help improve the sensitivity and accuracy of liquid biopsy testing^10–13^.

To further overcome the limitations of traditional mutation and fragmentation-based liquid biopsy testing, researchers have been exploring the use of cancer-specific methylation signals. This method involves analyzing altering patterns of DNA methylation that are characteristic of cancer cells and uses these features to detect the presence of cancer-specific methylation signals in cfDNA. This approach has been showing very promise as a more sensitive and accurate way to detect cancer, particularly in its early stages, and is an active area of research in the field of liquid biopsy testing^14–19^. Cancer-specific DNA methylation patterns are found as normal cells undergo transformation into cancer cells, with each type of cancer exhibiting unique methylation characteristics. By analyzing these patterns, liquid biopsy testing can potentially identify cancer-specific methylation signals and aid in the early detection and classification of cancer molecules^20–23^. As a result, detecting methylation in cfDNA may help not only confirm the presence of cancer but also differentiate between different types of cancer origin by identifying the cancer-specific DNA methylation pattern in different tissue types^24–26^.

**Figure 1:**
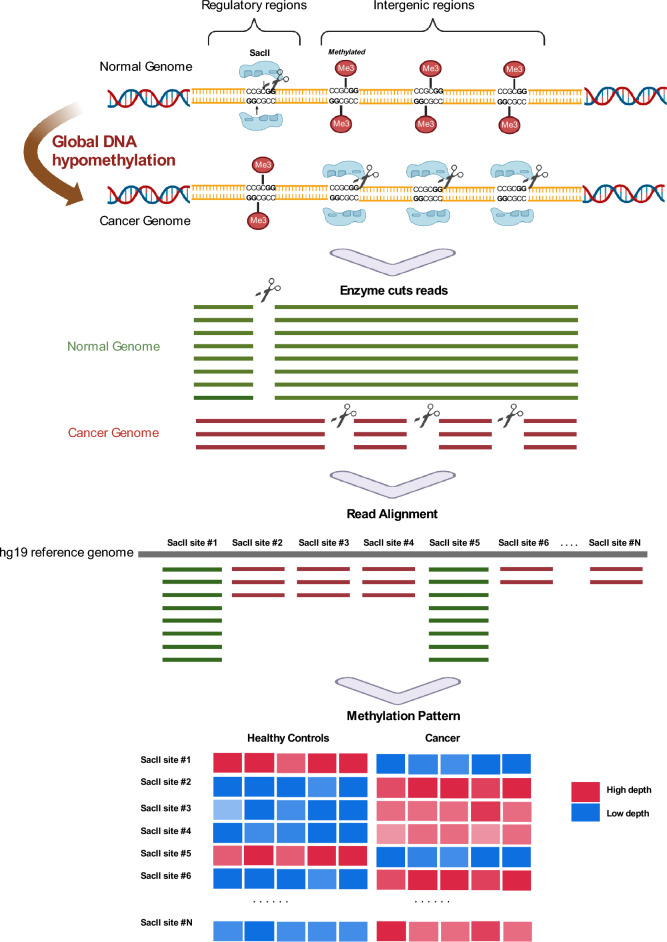
Methylation-sensitive restriction enzyme digestion followed by sequencing (MRE-seq) with a *Sac*II diagram. A library was constructed based on MRE-seq using a methylation-sensitive restriction enzyme, *Sac*II. As cancer grows, global DNA hypomethylation accelerates demethylation in both the regularity region and intragenic regions. In the cancer genome, demethylation occurs in differ regions, making a distinct pattern.

There are several types of methylation analysis in cancer cfDNA using NGS technology including bisulfite sequencing, affinity purification for methylated DNA and methylation sensitive restriction enzyme sequencing (MRE-seq). Although bisulfite sequencing is a widely used and effective method for methylation analysis in genomic DNA, it can be challenging to use with cfDNA from liquid biopsy samples due to several limitations, including fragmentation of cfDNA, low input DNA amount, DNA degradation and DNA methylation heterogeneity. Due to the limitations of bisulfite sequencing in analyzing cfDNA from liquid biopsy samples, there is a need for alternative cancer methylation enrichment sequencing technologies that can accurately detect cancer-specific methylation patterns in the presence of high levels of normal DNA background. MRE-Seq is a promising alternative approach to cancer cfDNA methylation analysis that involves cutting and capturing specific unmethylated sequences using a methylation-sensitive restriction enzyme. While MRE-Seq mostly has shown potential for detecting cancer methylation patterns in genomic DNA, it has not yet been widely studied for use in liquid biopsy testing because of the technical limitation in cfDNA analysis using existing methods^27,28^. The proposed MRE-Seq protocol in this article has the potential to be a highly sensitive and effective liquid biopsy method for detecting cancer-specific DNA methylation patterns in liquid biopsy for the following reasons. First, the MRE-Seq can analyze global hypomethylation in cancer cells, a characteristic feature of cancer genomes, by selectively cutting and sequencing unmethylated restriction sites in cancer DNA^29,30^. This process enriches ctDNA molecule and enhances sensitivity, which can improve the accuracy of early cancer detection (Fig. 1). Second, the MRE-Seq has a lesser impact on DNA degradation during sample preparation compared to bisulfite conversion, allowing for robust analysis using a relatively small amount of cfDNA^31,32^.

Next generation sequencing (NGS) was performed using MRE-Seq, which involves using the methylation-sensitive restriction enzyme *Sac*II to capture and sequence unmethylated restriction sites in cfDNA. As cancer develops, global DNA hypomethylation occurs, leading to accelerated demethylation in both regulatory and intragenic region of the genes. In the cancer genome, this demethylation occurs in distinct regions, leading to a specific methylation pattern that can be detected using MRE-Seq. By analyzing this pattern, it is possible to diagnose and detect the development of cancer using cell-free DNA by liquid biopsy analysis.

We investigate the utility of the proposed MRE-Seq method in diagnosing various cancers by analyzing liquid biopsy samples from patients with lung and colorectal cancers. Lung and colorectal cancer are the two leading causes of global cancer-related deaths^33^. Although the 5-year survival rates among patients with late-stage lung cancer and colorectal cancer remain below 20% and 14%, the survival rate can be increased to 70% and 90% if it is detected in early-stage of the cancer, respectively^34–37^. Although existing cancer screening analysis such as low-dose computed tomography (LDCT) is a commonly used for the early detection of lung cancer, it has high rate of false-positive results and risk of radiation exposure for patients^38^. While fecal occult blood tests and colonoscopies are currently recommended for the early detection of colorectal cancer, both methods have limitations. Fecal occult blood test is relatively simple procedure but less accurate than colonoscopies, and colonoscopies can be inconvenient and invasive for patients^39^. In order to overcome the limitation and disadvantage of the current cancer screening methods, liquid biopsy of cfDNA analyses has been explored as a safer, more convenient, and potentially more effective alternative for early cancer detection.

In this prospective study, the diagnostic performance of the new MRE-Seq method was evaluated for the detection of the cancers, and classification of the cancer signal origin (CSO) using a deep neural network (DNN) analysis. The aim of the study was to determine the accuracy of MRE-Seq for detecting cancer-specific DNA methylation patterns in cfDNA from liquid biopsy samples, and to investigate the potential of DNN method for detecting presence of cancer and identifying the type of cancer tissue. By exploring the diagnostic potential of the MRE-Seq and DNN, this study may contribute to the development of more accurate and effective methods for early cancer screening and diagnosis.

## Methods

### Study subjects

Treatment-naïve and histologically confirmed patients with lung cancer and colorectal cancer were enrolled in this study at Samsung Medical Center and Bucheon St. Mary’s Hospital, respectively. Patients with a history (within five years) of other malignancy were not included. For healthy controls, participants with no history of cancer diagnosis were enrolled at Gangnam Major Hospital.

### cfDNA library construction for MRE-seq

Eight-mL tubes of whole blood were collected (NICE® cfDNA tube) (EDGC, South Korea), which were centrifuged at 1900×*g* for 10 min and 13,000×*g* for 5 min for plasma separation. Samples with a hemoglobin level of ≤ 100 mg/dL were used in further analysis. The separated plasma was stored at − 70 °C until use.

CfDNA was extracted using 3.5–4 mL of plasma with the chemagic cfNA 5k Kit special H24 (Perkin Elmer) using chemagic™ 360 instrument according to the product manual. Extracted cfDNA is purified using HiAccuBead (Accugene) with 2X. CfDNA concentration was measured with a Qubit 2.0 fluorometer (Thermo Fisher Scientific). The extracted cfDNA was stored − 20 °C until use.

cfDNA (10–25 ng) was used for end-repair and A-tailing. Then, a p7 adapter with 10-bp unique molecular index (UMI) was ligated to the cfDNA with T4 DNA ligase (NEB, USA) at 3 µM at 25 °C for 2 h. After that, the p7-ligated cfDNA was treated with *Sac*II and ligated to a p5 adapter which have a cohesive end of *Sac*II digestion. PCR amplification was performed with 11 cycles using the p7 universal primer (5′-CAA GCA GAA GAC GGC ATA CGA-3′) and p5 universal primers (5′-AAT GAT ACG GCG ACC ACC GA-3′) with Taq DNA polymerase (Supplementary Table 1). Finally, size selection of PCR-amplified libraries between 200 and 550 bp was performed using PippinHT (Sage Science, USA). High-throughput NGS was performed using an Illumina Novaseq6000 sequencer with 100 PE (Supplementary Table 2).

### Data processing

NGS data were obtained in binary base call (BCL) sequence file format and converted to Fastq format using bcl2fastq v2.20. The sequenced read quality was examined with FastQC^40^ after removing reads shorter than 20 bp, single-end reads. The UMI sequence located at the beginning of R2 reads was used for deduplication. Reads containing even a single N or Q0 base in the UMI sequence were dropped during the quality-trimming step. BWA-MEM 0.7.15^41^ was used to align the processed Fastq sequences to the hg19 human reference genome and convert into binary alignment map (BAM) file format. In-house software removed PCR duplications and corrected sequencing errors using UMI sequence tags.

There are 67,472 *Sac*II site in the hg19 human genome and 63,266 *Sac*II sites of autosomal chromosomes, excluding sex chromosomes, were used as markers for analysis. For downstream analysis, the deduplicated read depth of each *Sac*II site was normalized by a trimmed mean, which was obtained by calculating the average depth of the total *Sac*II sites excluding 10% outliers (Fig. S1).

### Deep learning modeling

We implemented the multi-layer feed-forward neural network consisting of two hidden layers between the input and output layers. The normalized depth values corresponding to the 63,266 *Sac*II sites entered the input layer and went through two hidden layers consisting of 64 nodes with a Rectified Linear Unit activation function. Training was accomplished by calculating the loss of the cost function in the final output layer. The weight value of each node was updated by calculating the cross-entropy loss through the SoftMax activation function of the output layer and by performing backpropagation with 120 epochs toward a decreased value (Fig. S2). To build an accurate and robust prediction model, the dataset was split into training, testing, and validation sets. The training set encompassed the data sample used to fit the model, whereas the validation set was used to fine-tune the hyperparameters, i.e., the number of layers and nodes and batch and epoch sizes. The model was trained with the best parameters, and the test dataset was evaluated (Supplementary Methods, Fig. S3).

### Ethics approval and consent to participate

Approvals were obtained from the institutional review boards (IRBs) at the Samsung Medical Center (IRB: SMC 2019-11-080), Bucheon St. Mary’s Hospital (IRB: HC17TOSI0032), and Gangnam Major Hospital (IRB: DR_CPLX_001). Written informed consent was obtained from each study participant before enrollment. This study was conducted in accordance with the Declaration of Helsinki.

## Results

### Study participants

Whole blood samples were collected from 327 participants consisting of 102 with colorectal cancer, 99 with lung cancer, and 126 healthy controls. After excluding 6 patients who withdrew consent to participate and two patients with QC-failed samples, the final analysis included 96 patients with colorectal cancer, 95 with lung cancer, and 126 healthy controls for model training and performance evaluation. Colorectal cancer cohort was composed of 74 colon cancer samples and 22 rectal cancer samples and lung cancer cohort was composed of 86 non-small-cell lung cancer (NSCLC) samples and 9 small-cell lung cancer (SCLC) samples (Table 1).

**Table 1:** Clinical characteristics and demographics of patients with cancer and healthy controls.

	Patients with colorectal cancer (N = 96)	Patients with lung cancer (N = 95)	Healthy controls (N = 126)
Age, years	66 (58–75)	66 (60–71)	64 (56–68)
Sex			
Female	39 (40.6)	31 (32.6)	85 (67.5)
Male	57 (59.4)	64 (67.3)	41 (32.5)
BMI, kg/m^2^	23.5 (21.0–25.8)	23.1 (21.4–25.6)	N/A
Smoking			
Never smoker	N/A	32 (33.7)	N/A
Ex-smoker	N/A	7 (7.4)	N/A
Current smoker	N/A	56 (59.0)	N/A
Amount, pack-year	N/A	30 (15–45)	N/A
Tumor size, mm	35 (25.5–55)	32 (21–52)	N/A
Stage^1^			
I	17 (17.7)	32 (33.7)	N/A
II	21 (21.9)	9 (9.5)	N/A
III	46 (47.9)	23 (24.2)	N/A
IV	12 (12.5)	31 (32.6)	N/A
Histological type^2^			
Adenocarcinoma	95 (99.0)	61 (64.2)	N/A
Squamous cell carcinoma		20 (21.1)	N/A
Small cell		9 (9.5)	N/A
Other cell type	1 (1.0)	5 (5.3)	N/A
Tumor differentiation			
Well	9 (9.4)	2 (2.1)	N/A
Moderate	76 (79.2)	35 (36.8)	N/A
Poor	7 (7.3)	23 (24.2)	N/A
Missing or N/A	4 (4.2)	35 (36.8)	N/A
Vascular invasion			
Present	68 (70.8)	N/A	N/A
Absent	28 (29.2)	N/A	N/A
MSI			
MSS	86 (89.6)	N/A	N/A
MSI-H	7 (7.3)	N/A	N/A
Missing	3 (3.1)	N/A	N/A
Locations			
	Ascending colon: 20 (20.8)	RUL: 25 (26.3)	N/A
	Transverse colon: 5 (5.2)	RML: 8 (8.4)	N/A
	Descending colon: 49 (51.0)	RLL: 18 (19.0)	N/A
	Rectal: 22 (22.9)	LUL: 28 (29.5)	N/A
		LLL: 16 (16.8)	N/A

Data are presented as numbers (%) or the median (interquartile range).

*BMI*, body mass index; *MSI*, microsatellite instability; *MSS*, microsatellite stable; *MSI-H*, high microsatellite instability; *N/A*, not applicable;

*NSCLC*, non-small-cell lung cancer; *RLL*, right lower lobe; *RML*, right middle lobe; *RUL*, right upper lobe; *LLL*, left lower lobe; *LUL*, left upper lobe; *SCLC*, small-cell lung cancer.

^1^NSCLC (*N* = 86) and SCLC (*N* = 9) were staged according to the 8^th^ edition of the American Joint Committee on Cancer.

^2^One patient in colorectal cancer group had neuroendocrine carcinoma. Other NSCLCs (*N* = 5) included large-cell neuroendocrine carcinoma (*N* = 3), adenosquamous cell carcinoma (*N* = 1), pleomorphic carcinoma (*N* = 1), and NSCLC not otherwise specified (*N* = 1).

### MRE-seq of cfDNA

*Sac*II, a methylation-sensitive restriction enzyme, was used for MRE-seq-based liquid biopsy in this study. Approximately 90% of reads produced by MRE-seq were mapped to hg19 reference genome. After deduplication based on the UMI, the remaining read ratio was 42–52% compared with the original mapped reads. The mapping coverage at a depth of at least one *Sac*II site ranged between 96 and 99% of the 63,266 target sites. Among the deduplicated reads, those with the 5′ end “GCGG” sequence matching the *Sac*II cut site were defined as on-target reads, and the ratio of on-target reads to deduplicated reads is defined as the on-target ratio. The on-target ratio of samples ranged 50–57% which was no significant difference between colorectal cancer, lung cancer, and healthy controls (Fig. S4).

The heatmap plot of the top 1,000 markers from each cancer types showed distinguishable patterns with high statistical power (student t-test *P* < 1 × 10^−7^) for differentiating cancer from healthy controls. (Fig. S5, Supplementary Table 3A,B).

Among the 63,266 target sites, most of the *Sac*II sites were uniformly distributed in intron regions (31.0%; 19,649), the promoter (25.7%; 16,285) and intergenic regions (24.8%; 15,699), followed by exons, 5’ UTR in 5,871 (9.3%) and 2,240 (3.5%) cases, respectively, which is suitable for global hypomethylation analysis. SHapley Additive exPlanations (SHAP)^42^ assigns each feature an importance value after the model training (Supplementary Table 4A,B). The top 1,000 markers with high feature importance were obtained with SHAP from our DNN model, and these markers were also evenly distributed in regulatory and intergenic regions (Fig. S6, Supplementary Table 5).

### Evaluation of prediction performance of DNN model

We defined the probability value of output layer of DNN model as a cancer score. We performed 20 independent repetitions of nested fivefold cross-validation which makes 100 different cancer scores per each sample and used the average of cancer scores to assess the performance of our DNN model. In each cross-validation cycle, a classification model was trained and test samples which were excluded from the training set were evaluated (Fig. S3). The interquartile range (IQR) was also calculated to measure how stable the scores of test sample are in various models.

The average IQR values of cancer score was 0.09 for cancer samples and 0.06 for the healthy control samples in the colorectal cancer classification model. In the lung cancer classification model, the average IQR of cancer samples and the healthy control samples were 0.13 and 0.10, respectively (Fig. S7). Therefore, the cancer scores appeared consistent for each cross-validation cycle. Additionally, to check whether the number of samples is sufficient to evaluate model performance, area under the receiver operating characteristic curve (AUC) and the average IQR were measured by randomly selecting samples with different sample size ratios. In the colorectal cancer model, reducing the number of samples by 50% only decreased the AUC by 0.02 and increased the average IQR by 0.015. (Fig. S8a,b). In the lung cancer model, AUC is almost saturated from the sample size ratio of 60%, and the average IQR showed only 0.03 difference in the sample size ratio of 50%. (Fig. S8c, d).

### Colorectal cancer classification

**Figure 2:**
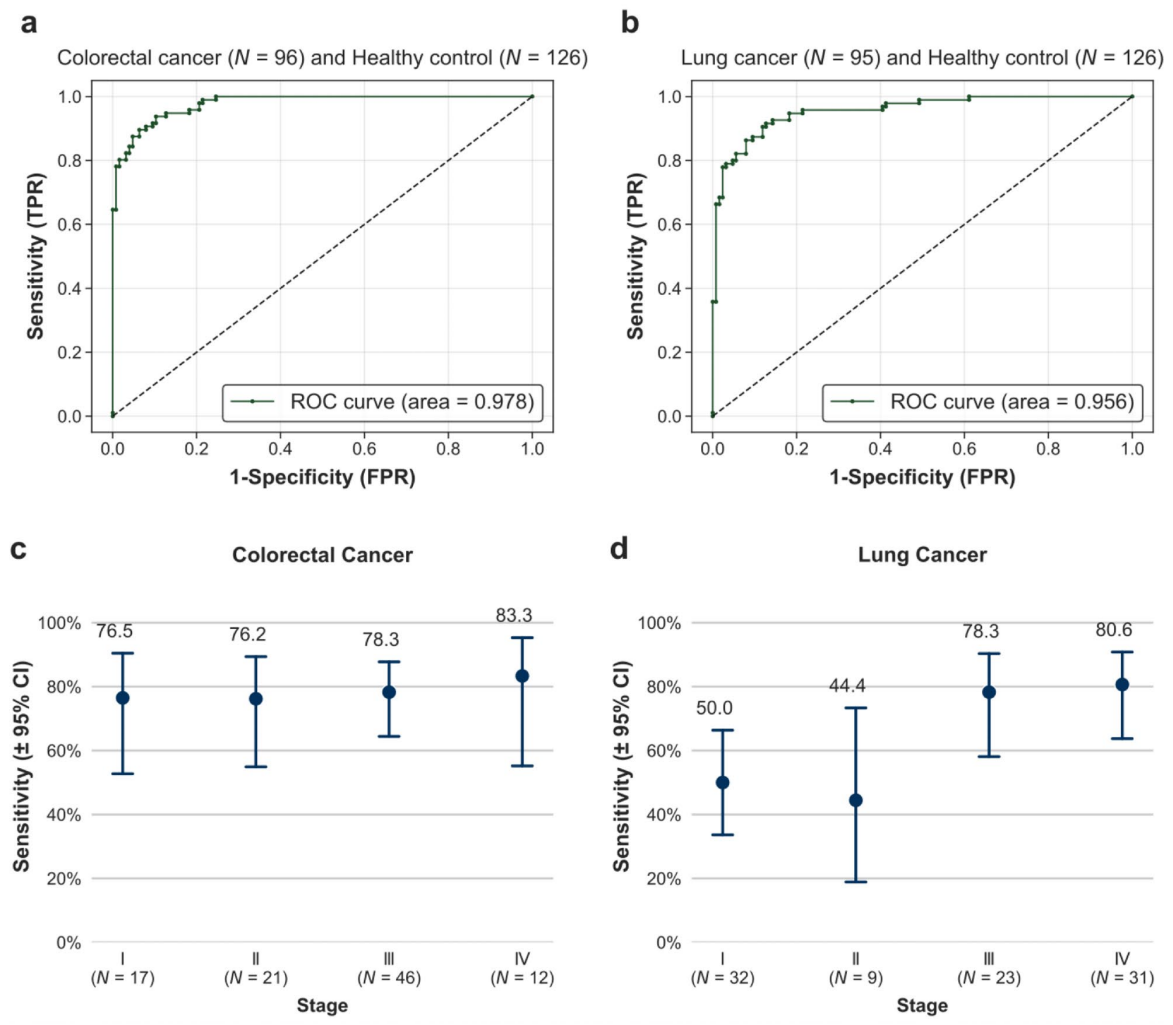
Test performance of Colorectal and Lung cancer classification. (**a**,**b**) The overall AUC values were 0.978 for colorectal cancer and 0.956 for lung cancer. (**c**,**d**) Sensitivity at 99.2% specificity with 95% confidence interval (CI) according to cancer stage.

The AUC was 0.978, and the overall sensitivity was 78.1% (95% confidence interval [CI] 68.9–85.2%), with 76.5% (95% CI 52.7–90.4%), 76.2% (95% CI 54.9–89.4%), 78.3% (95% CI 64.4–87.7%), and 83.3% (95% CI 55.2–95.3%) sensitivity for stage I, II, III, and IV, respectively, at 99.2% specificity (Table 2, Fig. 2, Supplementary Table 6, Fig. S9).

**Table 2:** Sensitivity of the DNN model for predicting colorectal cancer and lung cancer at a specificity of 99.2%.

Healthy controls						
	N	Positive	Specificity (95% CI)			
Total	126	1	99.2 (95.6–99.9)*			

Clinical stage	Colorectal cancer			Lung cancer		
	Total *N*	Positive	Sensitivity (95% CI)	Total *N*	Positive	Sensitivity (95% CI)
All	96	75	78.1 (68.9–85.2)	95	63	66.3 (56.3–75.0)
I	17	13	76.5 (52.7–90.4)	32	16	50.0 (33.6–66.4)
II	21	16	76.2 (54.9–89.4%)	9	4	44.4 (18.9–73.3)
III	46	36	78.3 (64.4–87.7)	23	18	78.3 (58.1–90.3)
IV	12	10	83.3 (55.2–95.3)	31	25	80.6 (63.7–90.8)
I–II	38	29	76.3 (60.8–87.0)	41	20	48.8 (34.3–63.5)

*Sensitivity for each cancer type was calculated based on the specificity of 99.2%, which allowed 1 false positive sample out of 126 healthy control samples.

**Figure 3:**
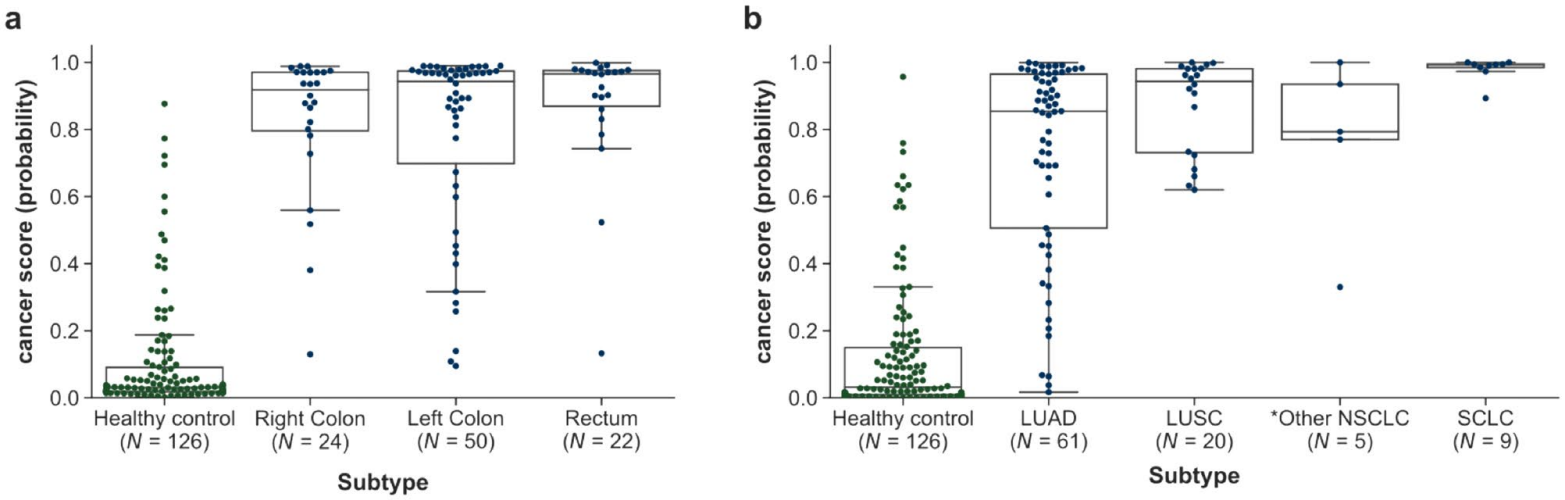
Cancer score distribution according to subtype. (**a**) Colorectal cancer subtypes: right, left, and rectum. (**b**) Lung cancer subtypes: NSCLC and SCLC. *Right colon (cecum, ascending colon, hepatic flexure colon, and traverse colon), left colon (splenic flexure colon + descending colon + double S colon + sigmoid colon), rectum (rectosigmoid colon + rectum). * One patient in colorectal cancer group had neuroendocrine carcinoma. Other NSCLCs (*N* = 5) include large-cell neuroendocrine carcinoma (*N* = 3), adenosquamous cell carcinoma (*N* = 1), pleomorphic carcinoma (*N* = 1), and NSCLC not otherwise specified (*N* = 1).

Among the 21 false-negative samples, 18 were colon cancers with an FNR of 24% (18/74), and 3 samples were rectal cancers with an FNR of 13.6% (3/22). Among these 18 false-negatives, 61.1% (11/18) were from the left colon, which comprises the left half of the transverse colon, splenic flexure, descending colon, and sigmoid colon (Fig. 3a).

### Lung cancer classification

The AUC was 0.956, and overall sensitivity of 66.3% (95% CI 56.3–75.0%), with 50.0% (95% CI 33.6–66.4%), 44.4% (95% CI 18.9–73.3%), 78.3% (95% CI 58.1–90.3%), and 80.6% (95% CI 63.7–90.8%) sensitivity for stage I, II, III, and IV, respectively, at 99.2% specificity. The sensitivity for SCLC was 100.0% (95% CI 43.9–100%) in limited disease and 100.0% (95% CI 61.0–100%) in extensive disease, which was better than those obtained for NSCLC (Table 2, Fig. 2, Supplementary Table 6, Fig. S9).

NSCLC has a heterogeneous histological type and is divided into LUAD and lung squamous cell carcinoma (LUSC). As shown in Fig. 3b, LUSC had a significantly higher cancer score than LUAD (*P* = 0.030). Additionally, all nine SCLCs had 100% sensitivity with a very high cancer score (0.98 on average for SCLC) (Fig. 3b).

### CSO prediction

**Figure 4:**
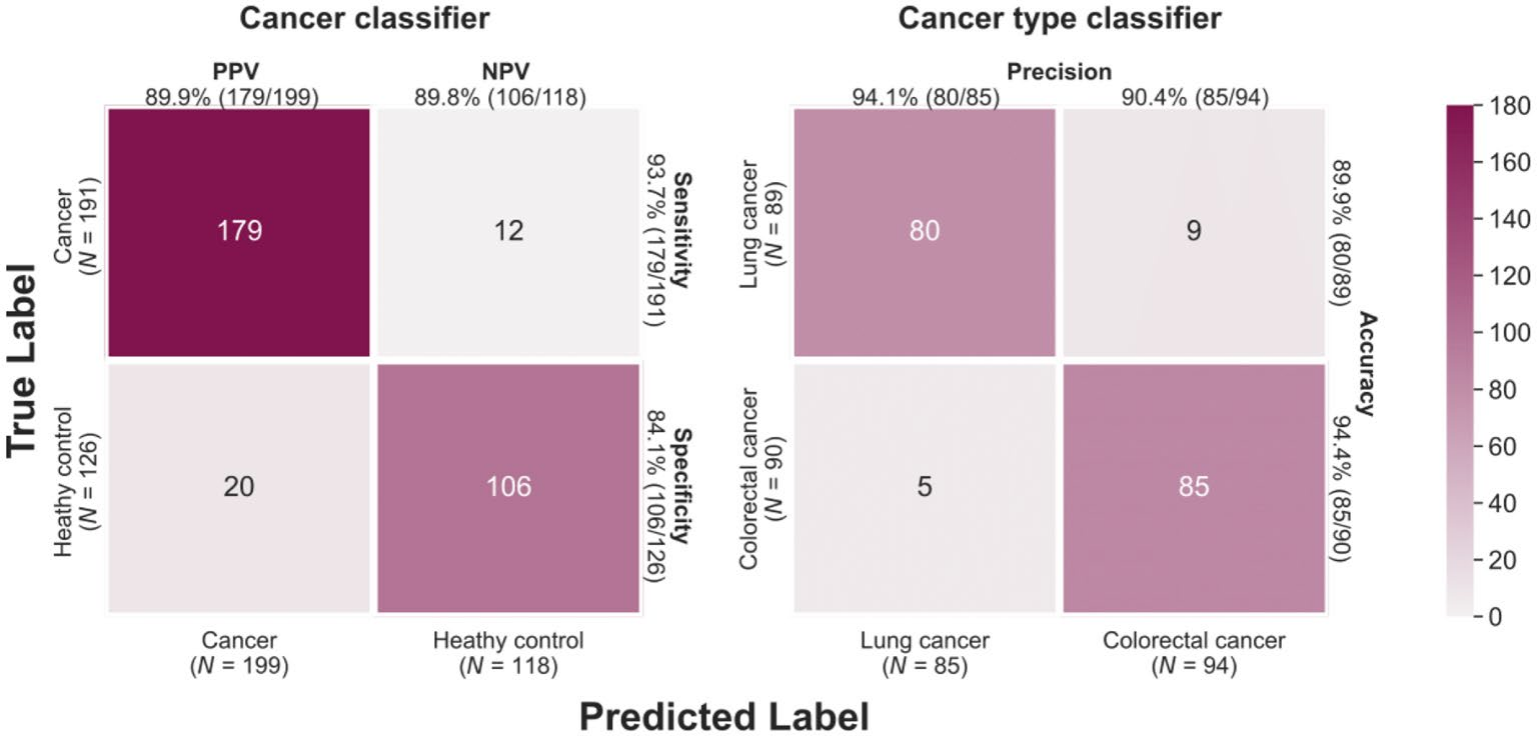
Confusion matrix of the cancer signal origin (CSO). CSO accuracy was measured using two different classifiers: the Cancer Classifier for determining cancer existence and the Cancer Type Classifier for identifying the type of cancer.

CSO prediction model consists with two classifiers: the Cancer Classifier, which determines whether cancer is present, and the Cancer Type Classifier, which classifies the type of cancer. Prediction performance was measured through fivefold cross-validation which samples 80% of the data for training and 20% for testing. In each fold, the Cancer Classifier was trained using two cancer type samples as a case group and the healthy controls as a control group. Afterwards, the true positives were tested in the Cancer Type Classifier which was built using two cancer types with different labels. A cancer type with the highest probability value was defined as a true positive. The accuracy of these two classifiers was displayed in the confusion matrix (Fig. 4). In the Cancer Classifier, 179 out of 191 cancer samples were positively predicted with a sensitivity of 93.7% and they were classified into the two cancer types using the Cancer Type Classifier with high accuracy (94.4% in colorectal and 89.9% in lung cancer).

### Confounder analysis

Even after normalizing the data, principal component analysis (PCA) is commonly used to determine whether sequencing batch effects exist^43,44^. It has been confirmed with PCA that there was no bias between the 22 batches or between the sample groups. (Fig. S10). Seven samples were identified as outliers if Principal component 1 (PC1) exceeded 250, and they were over stage 3 cancer samples with high cancer scores.

Considering that methylation changes are affected by age^45^, it is possible that age becomes a confounding factor if the age distribution differs between the sample groups. In this study, there was a statistically significant difference in age distribution (student t-test *P* = 0.003 for colorectal cancer vs. healthy controls and *P* = 0.022 for lung cancer vs. healthy controls). However, the age was not correlated with the cancer score. The Pearson's correlation coefficient (PCC) between cancer score and age was 0.005 for colorectal cancer samples and 0.096 for lung cancer samples. For the normal group, the PCC values in the colorectal cancer classification model and lung cancer classification model were 0.071 and 0.061, respectively. The distribution of cancer scores was not significantly different among the age groups (Fig. S11a).

Because the dataset contained males and females, cancer-related markers on the sex chromosomes may lead to incorrect analytical results. To avoid this, all analyses were carried out using only markers on autosomal chromosomes. Still, if there is a large difference in the cancer scores between males and females, sex may act as a confounding factor. In the colorectal cancer model, both genders showed similar distributions, but in the lung cancer model, male patients showed a significantly higher cancer score (Fig. S11b). To address this sex difference, we compared the characteristics of patients with lung cancer by sex. As shown in Supplementary Table 7, 87.1% (27/31) of the female patients were never-smokers, whereas 91.2% (59/64) of the male patients were current or former smokers. Moreover, the female patients in the lung cancer group were significantly younger and had a higher prevalence of LUAD and early stages compared with the male patients. Because these factors (age, smoking, histology, and cancer stage) might have confounded the results, we performed multivariable analysis (Supplementary Table 8) and found that smoking was an independent factor associated with lung cancer score (Fig. S12). All tests were two-sided, and significance was set at *P* < 0.05. We used Stata software (v. 14.0; Stata Corporation, College Station, TX, USA) for statistical analysis.

## Discussion

This study presents a novel liquid biopsy method for cancer detection using the proposed MRE-Seq method and a DNN artificial intelligence (AI) analysis. The method was found to be highly sensitive and accurate in detecting cancer-specific DNA methylation patterns in cfDNA and has the potential to be a valuable tool for early cancer diagnosis and detection.

In recent years, analysis of the methylation pattern in cfDNA has emerged as a promising cancer screening and monitoring protocol for the development of multicancer liquid biopsy methods^46–48^. The bisulfite sequencing was the most extensively studied method for analyzing DNA methylation in cancer. It has been used in a recent study on 27 different types of cancer and found that the methylation analysis was highly accurate and showed outstanding results in 16 cancer types with specificity of 99.4% and a sensitivity of 60% to 94% with the 92% correct classification of the CSO, an important index for early diagnosis in clinical practice^18,19^. However, the bisulfite sequencing analysis is very challenging and difficult to adopt in routine clinical setting due to a requirement of high amount of input blood because 84–96% of the DNA is subject to degraded during the bisulfite conversion step^31,32,49–51^.

Methylated DNA immunoprecipitation coupled with high-throughput sequencing (MeDIP-seq) was employed as an affinity-purification-based method, with AUCs of 0.978, 0.918, and 0.971 for acute myeloid leukemia, pancreatic cancer, and lung cancer, respectively^52^. However, the overall CSO prediction accuracy was less than70%, which is insufficient to become a practical tool for early multicancer screening.

Our proposed MRE-seq performance is comparable to the previous bisulfite sequencing method in accuracy of the cancer detection and classification of CSO with use of relatively smaller amount of blood from a single tube collection, and require lower sequencing depth coverage compared to the whole genome bisulfite sequencing analysis. These features enhance its practicability for routine clinical adoption by lowering requirement of patient blood sample and reducing the cost of the testing^28,31,51^.

The overall accuracy of cancer detection by MRE-seq was high because MRE-seq measures global hypomethylation, a characteristic feature of most cancer genome. In particular, the sensitivity of stage 1 samples of colorectal and lung cancers was 76.5% and 50.0%, respectively. The high detection rate for the early-stage cancer may be due to a prevention of DNA damage by avoiding bisulfite treatment and enhancing cancer signal by enriching cancer-specific demethylated reads (hypomethylation). Therefore, this method is more suitable for diagnosing early cancer in liquid biopsy using a small amount of cfDNA in a regular clinical testing.

The overall accuracy of the liquid biopsy method was found to be lower for lung cancer than for colorectal cancer. This is likely due to the greater diversity of histological subtypes and larger differences in DNA methylation patterns in lung cancer compared to colorectal cancer. However, the accuracy of lung cancer detection is expected to improve with the use of a sufficient number of LUAD samples in the training set.

Although only two cancer types were used in the testing the feasibility of CSO analysis using the MRE-Seq and deep learning analysis was, the results showed that the method had high accuracy in predicting the tissue of origin for most of the samples. However, there were more falsely predicted samples in certain subtypes of cancers, such as left colon in colorectal cancer and LUAD in lung cancer. By analyzing a sufficient number of samples with a similar number of each subtype, the accuracy of the CSO analysis may be improved in future work (Fig. S10).

In the cfDNA of cancer patients, both cancer-specific methylation patterns of ctDNA and tissue-specific patterns can coexist. As a result, some methylation signals detected in liquid biopsy samples may be tissue-specific rather than cancer-specific. To distinguish between cancer-specific and tissue-specific methylation patterns, it is necessary to perform comparative analyses with samples from patients with benign diseases related to the cancer type. By analyzing the methylation patterns of both cancer and benign disease samples, it may be possible to develop more accurate and specific liquid biopsy methods for cancer diagnosis^53^. In this study, samples with benign disease were not excluded, which may reflect real-world situations more accurately. By including samples with both cancer and benign disease, the study may provide a more realistic assessment of the accuracy and reliability of the liquid biopsy testing.

The study has several limitations that should be considered when interpreting the results. First, each case study was conducted at a single center, which may introduce selection bias and limit the generalizability of the findings. Additional multicenter studies are needed to validate the results with the independent test samples and confirm the utility of the method in different populations. Second, no follow-up was conducted for the healthy controls, which may have led to misclassification bias if certain individuals developed cancer after the study. Further research is needed to track the health outcomes of the controls and to assess the long-term predictive power of the method. Third, the study only included two types of cancer samples, which may limit the accuracy of CSO classification, especially for cancers with similar methylation patterns. To improve the performance of CSO prediction, additional studies using larger and more diverse sets of cancer types are required. Despite these limitations, the study provides valuable insights into the potential of the MRE-seq and DNN analysis of liquid biopsy methods for early cancer detection and diagnosis and may facilitate more effective treatment of the disease.

## Conclusions

The study aimed to develop a screening method for early detection of multiple cancers using liquid biopsy-based testing. By combining the proposed MRE-Seq and machine learning algorithm, the researchers were able to detect and classify colorectal and lung cancers with high accuracy. The MRE-Seq allows for the analysis of global hypomethylation in cancer genomes with high sensitivity and low cost with small blood sample requirement, which makes it a promising approach for early screening of multiple cancer types in routine clinical setting. However, additional research is needed to adapt and validate the method for other cancer types, and to determine its clinical feasibility for multicancer early detection. The study highlights the potential of liquid biopsy methods for improving cancer diagnosis and detection, suggesting that further development and validation of these methods could have important implications for improving cancer survival and quality of life.

### Supplementary Information

Supplementary Information 1.

Supplementary Information 2.

## Data Availability

The datasets generated and/or analysed during the current study are available in the NCBI Sequence Read Archive (SRA) under BioProject ID (PRJNA954459), http://www.ncbi.nlm.nih.gov/bioproject/954459.

## Abbreviations

BAM: Binary alignment map
BMI: Body mass index
CI: Confidence interval
DNN: Deep neural network
EDGC: Eone-Diagnomics Genome Center
FNR: False-negative rate
LDCT: Low-dose computed tomography
LUAD: Lung adenocarcinoma
LUSC: Lung squamous cell carcinoma
NGS: Next-generation sequencing
NSCLC: Non-small-cell lung cancer
PCR: Polymerase chain reaction
CSO: Cancer signal origin
UMI: Unique molecular identifier
