# Supplementary material for: Advances in methylation analysis of liquid biopsy in early cancer detection of colorectal and lung cancer
Source: Sci Rep. 2023 Aug 19;13:13502. doi: 10.1038/s41598-023-40611-w (PMC10439900; doi:10.1038/s41598-023-40611-w)
Supplement: Supplementary file 2 — Supplementary Information 2. [file 41598_2023_40611_MOESM2_ESM.docx]

**Supplementary Figures, Tables, and Method for:**

**Advances in Methylation Analysis of Liquid Biopsy in Early Cancer Detection of Colorectal and Lung Cancer**

Hyuk-Jung Kwon^1^†, Sun Hye Shin^2^†, Hyun Ho Kim^3^†, Na Young Min^1^†, YuGyeong Lim^1^, Tae-woon Joo^1^, Kyoung Joo Lee^1^, Min-Seon Jeong^1^, Hyojung Kim^1^, Seon-young Yun^1^, YoonHee Kim^1^, Dabin Park^1^, Joungsu Joo^1^, Jin-Sik Bae^1^, Sunghoon Lee^1^, Byeong-Ho Jeong^2^, Kyungjong Lee^2^, Hayemin Lee^3^, Hong Kwan Kim^4^, Kyongchol Kim^5^, Sang-Won Um^2^*, Changhyeok An^3^*, Min Seob Lee^1,6^*

^1^R&D Department, Eone-Diagnomics Genome Center, Inc., 143 Gaetbeol-ro, Yeonsu-gu, Incheon, 21999, Republic of Korea

^2^Division of Pulmonary and Critical Care Medicine, Department of Medicine, Samsung Medical Center, Sungkyunkwan University School of Medicine, 81 Irwon-ro, Gangnam-gu, Seoul, 06351, Republic of Korea

^3^Department of Surgery, Bucheon St. Mary’s Hospital, College of Medicine, The Catholic University of Korea, 327 Sosa-ro, Bucheon, 14647, Republic of Korea

^4^Department of Thoracic and Cardiovascular Surgery, Samsung Medical Center, Sungkyunkwan University School of Medicine, 81 Irwon-ro, Gangnam-gu, Seoul, 06351, Republic of Korea

^5^Gangnam Major Hospital, 452 Dogok-ro, Gangnam-gu, Seoul, 06279, Republic of Korea

^6^Diagnomics, Inc., 5795 Kearny Villa Rd. San Diego, CA 92123, USA

† These four authors contributed equally to this article

^*^Corresponding authors:

Dr. Sang-Won Um,

Division of Pulmonary and Critical Care Medicine, Department of Medicine, Samsung Medical Center, Sungkyunkwan University School of Medicine, 81 Irwon-ro, Gangnam-gu, Seoul, 06351, Republic of Korea,

Phone No: +82-10-9933-1645

Email: sangwonum@skku.edu

Dr. Chang Hyeok An,

Department of Surgery, Bucheon St. Mary’s Hospital, College of Medicine, The Catholic University of Korea, 327 Sosa-ro, Bucheon, 14647, Republic of Korea

Phone No: +82-10-5127-5454

Email: achcolo@catholic.ac.kr

Min Seob Lee, Ph.D.

Eone-Diagnomics Genome Center, Inc., 291 Harmony-ro, Yeonsu-gu, Incheon, 22014, Republic of Korea

Phone No: +82-10-3080-1393

Email: mlee@edgc.com

**
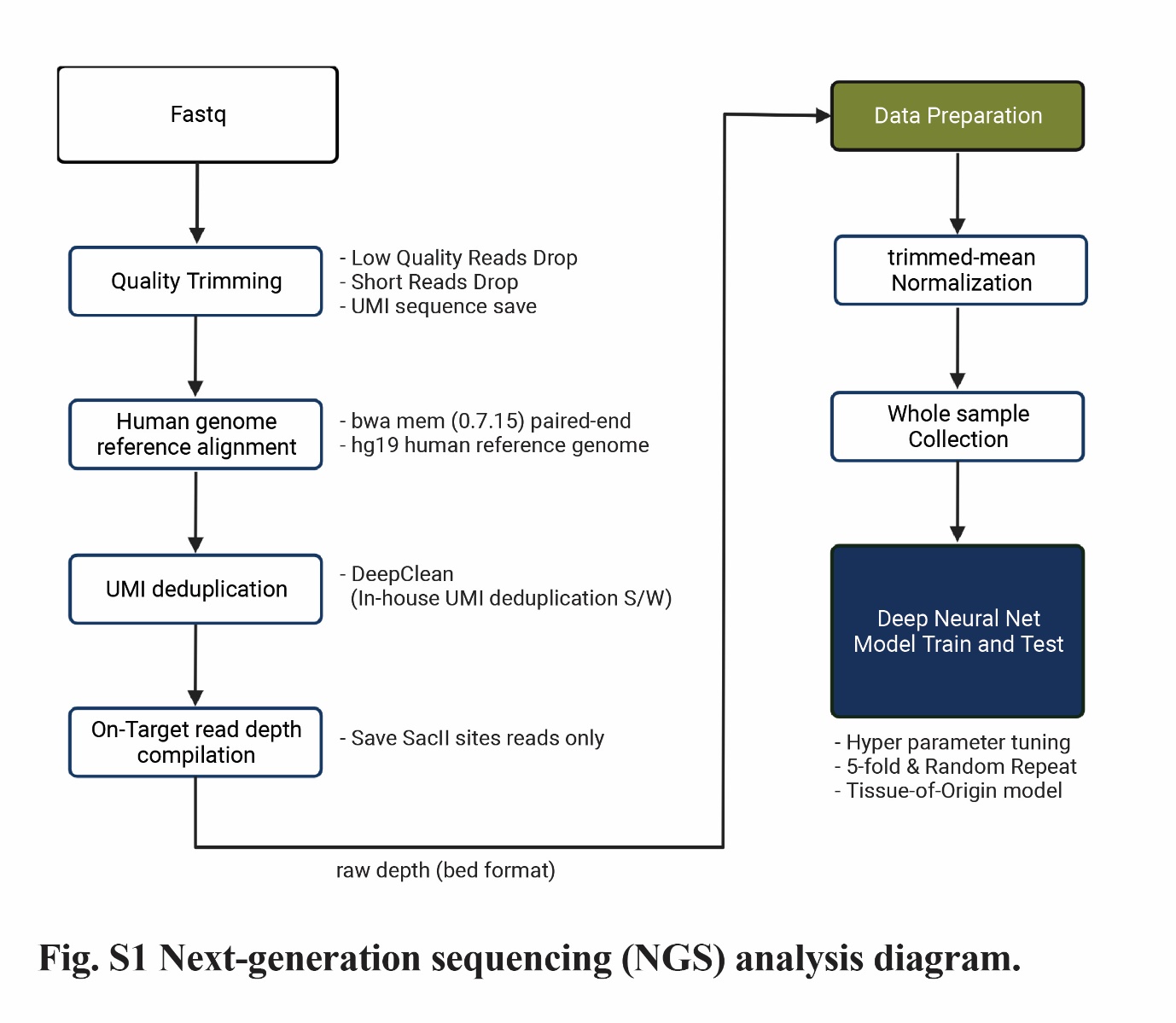
**

Fastq data were aligned to the hg19 human genome reference after quality trimming. Polymerase chain reaction (PCR) duplicated reads were removed using unique molecular identifier (UMI), and the on-target reads in *Sac*II sites were normalized to proceed with the downstream analysis and deep neural network (DNN) training


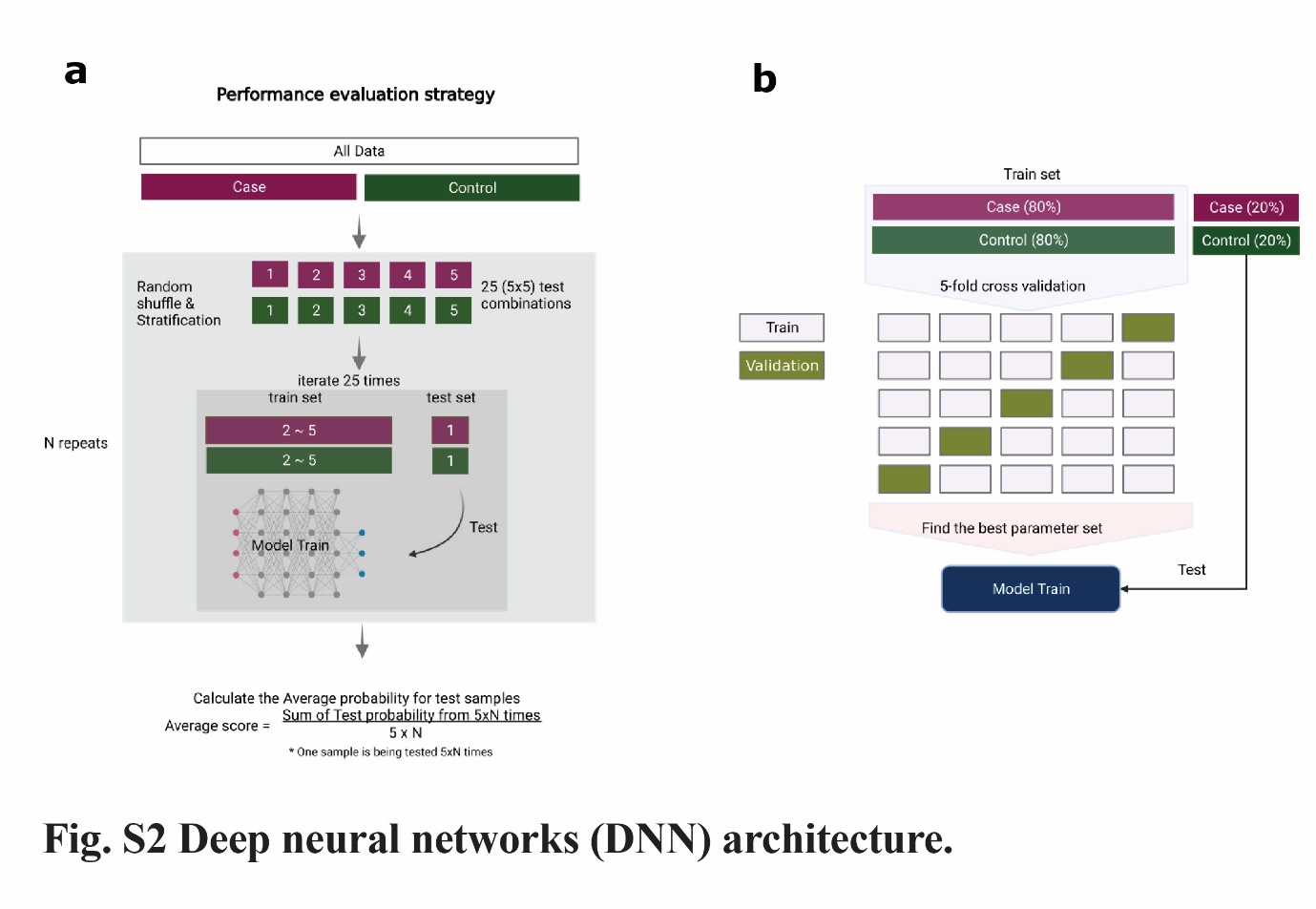


DNN consists of an input layer, an output layer, and many hidden layers in the middle. Each layer consists of neurons that have a weight value and the activation function. During the training process, each weight value is updated using a gradient descent algorithm and backpropagation


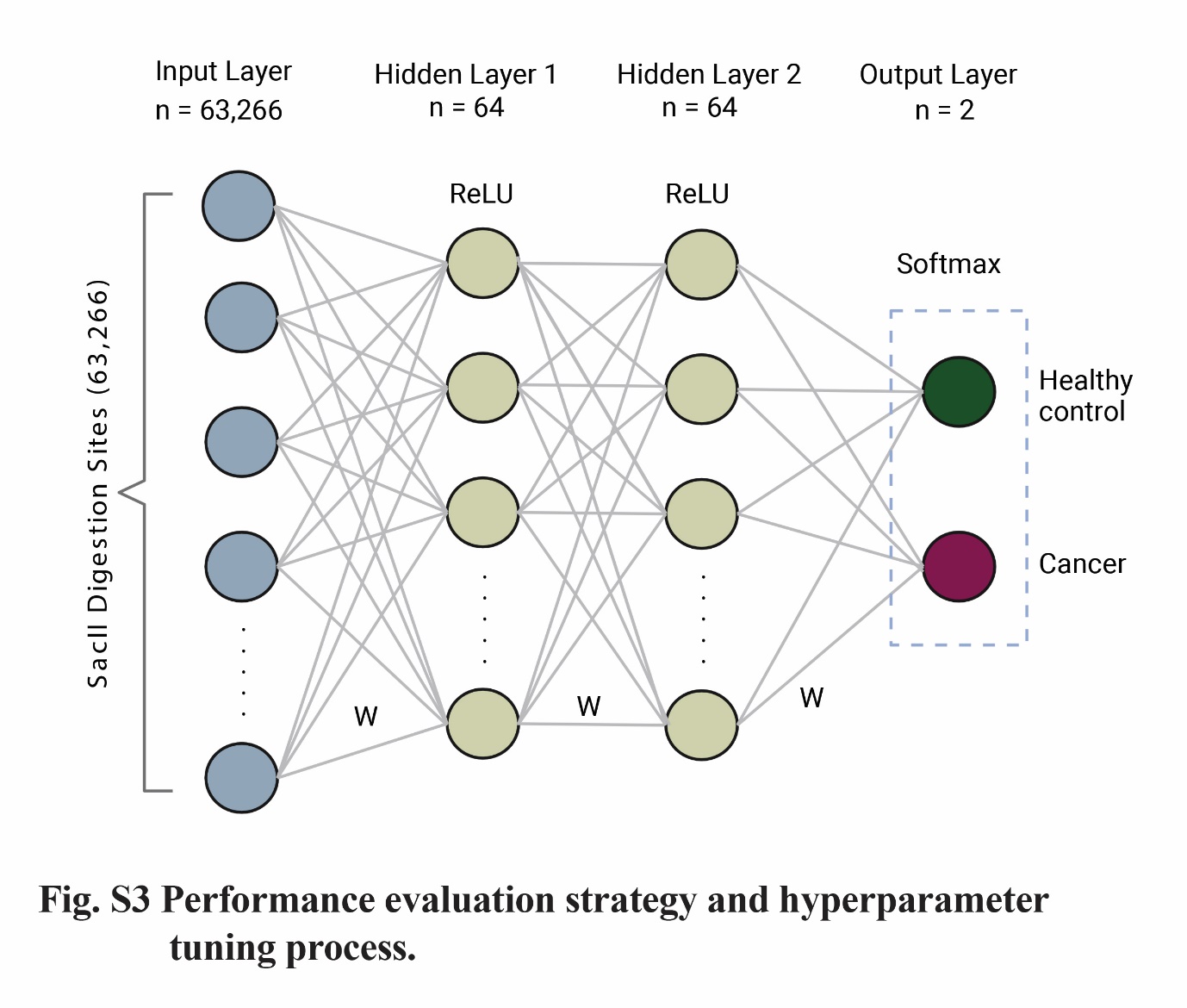


The number of nodes, hidden layers, epoch, and learning rate were tuned in each fold of the cross-validation (a). Nested K-fold cross-validation was used for reliable performance estimation. By performing 20 repetitions, a total of 500 different models were trained, and each sample was tested 100 times. (b) The most frequently observed hyperparameter set in the K-fold repetition was chosen as the best parameter

**
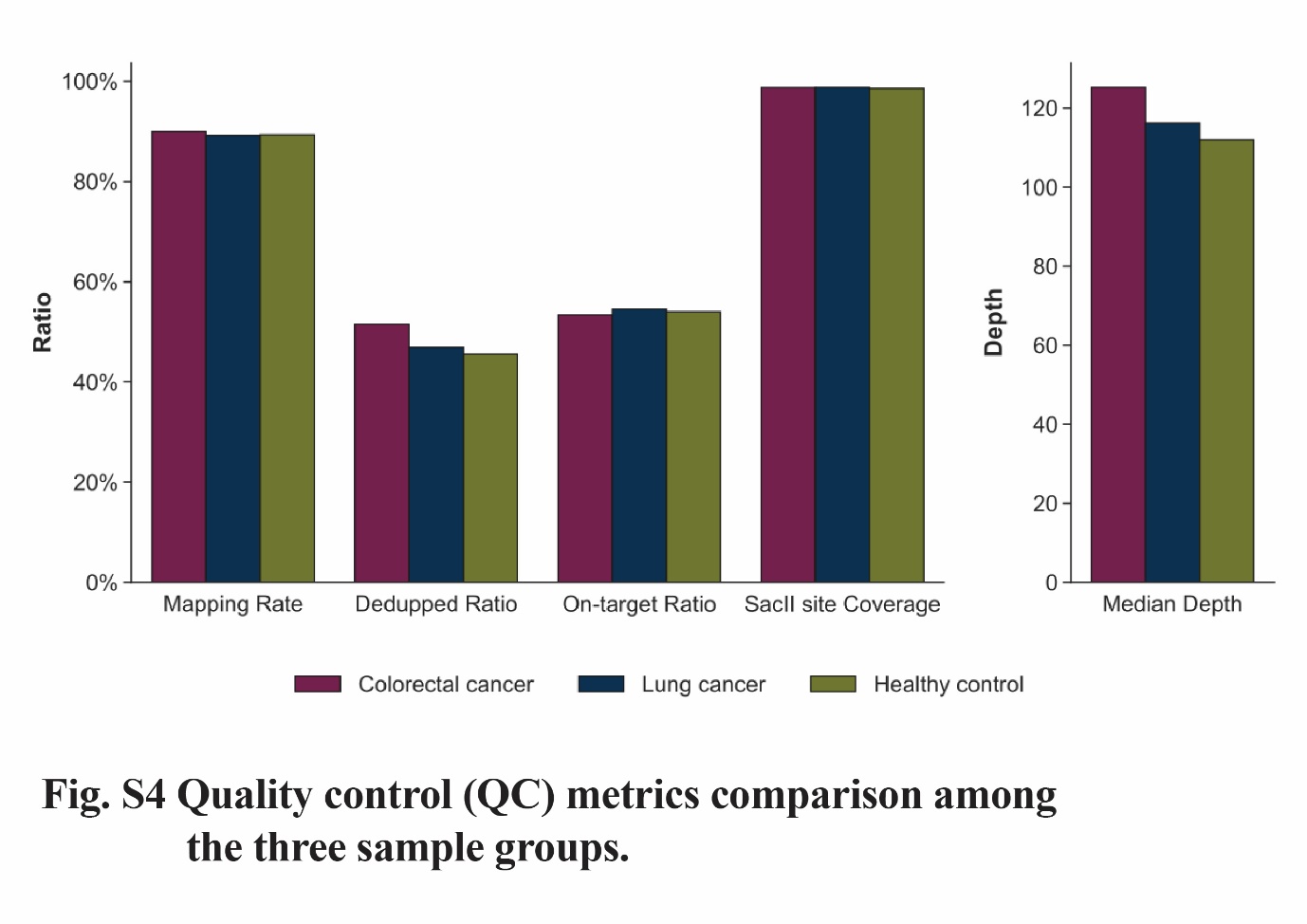
**

Mapping rate, on-target ratio, and *Sac*II site coverage were almost identical in the three groups. The median depth caused by the difference in the deduplicated ratio was negligible, although there was a slight difference between the three groups


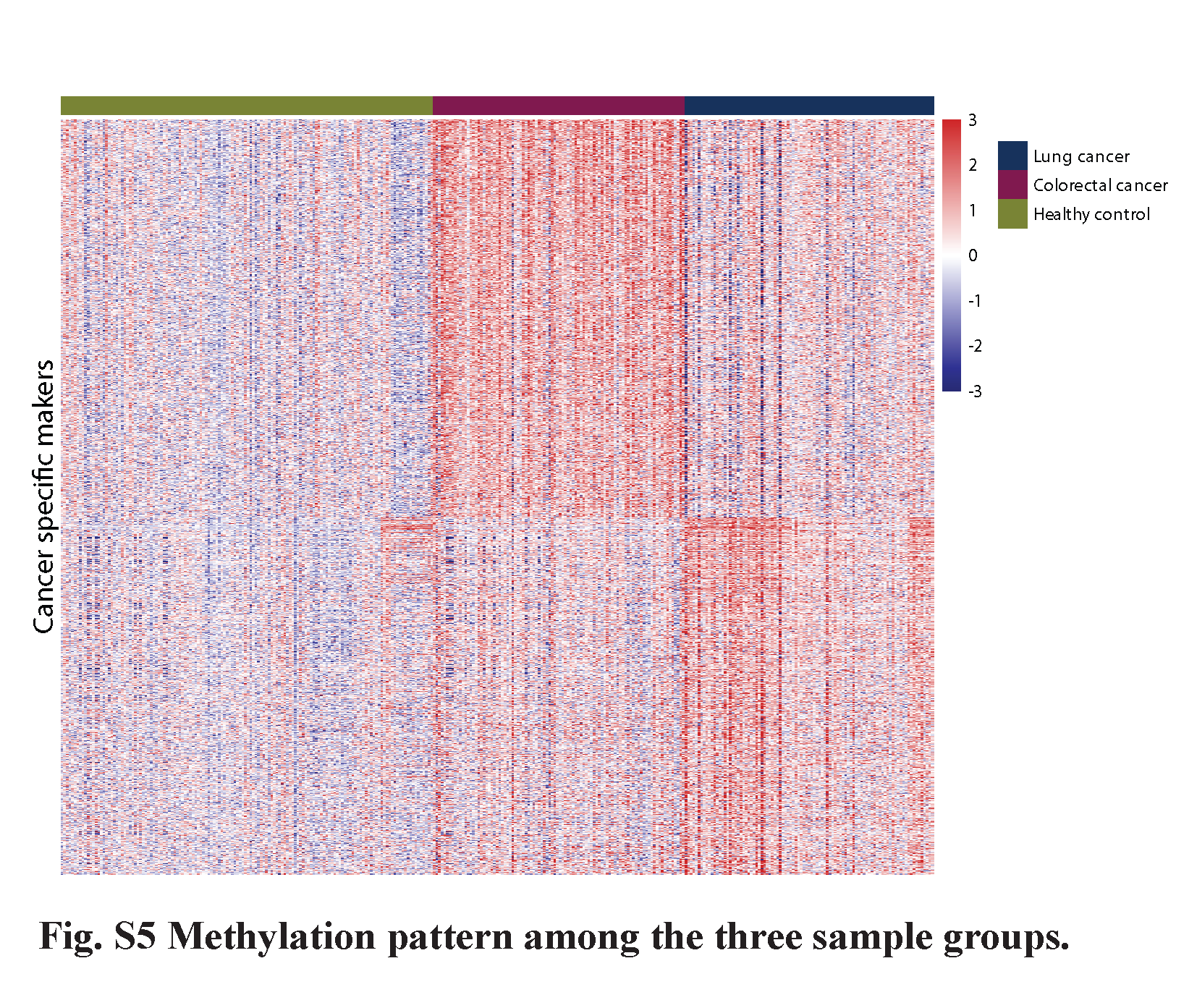


The heatmap of the three sample groups using the top 1,000 markers by student t-test for each cancer type. The colors on the top indicate the healthy controls, colorectal cancer, and lung cancer from left to right

**
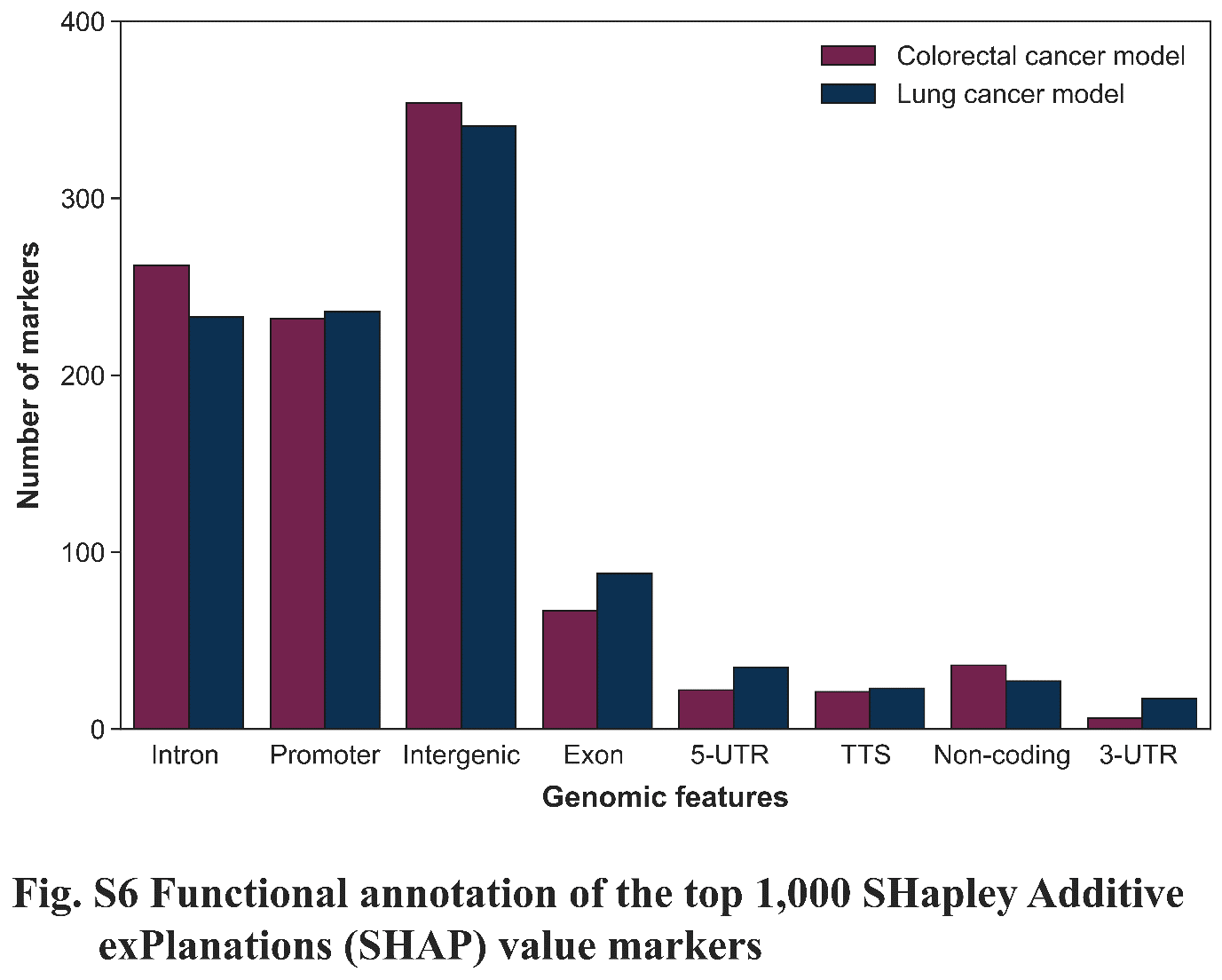
**

Top 1,000 SHAP-positive values with functional annotation. Among the 1,000 *Sac*II sites, most of the positive features were in regulatory and global regions with almost equal proportion


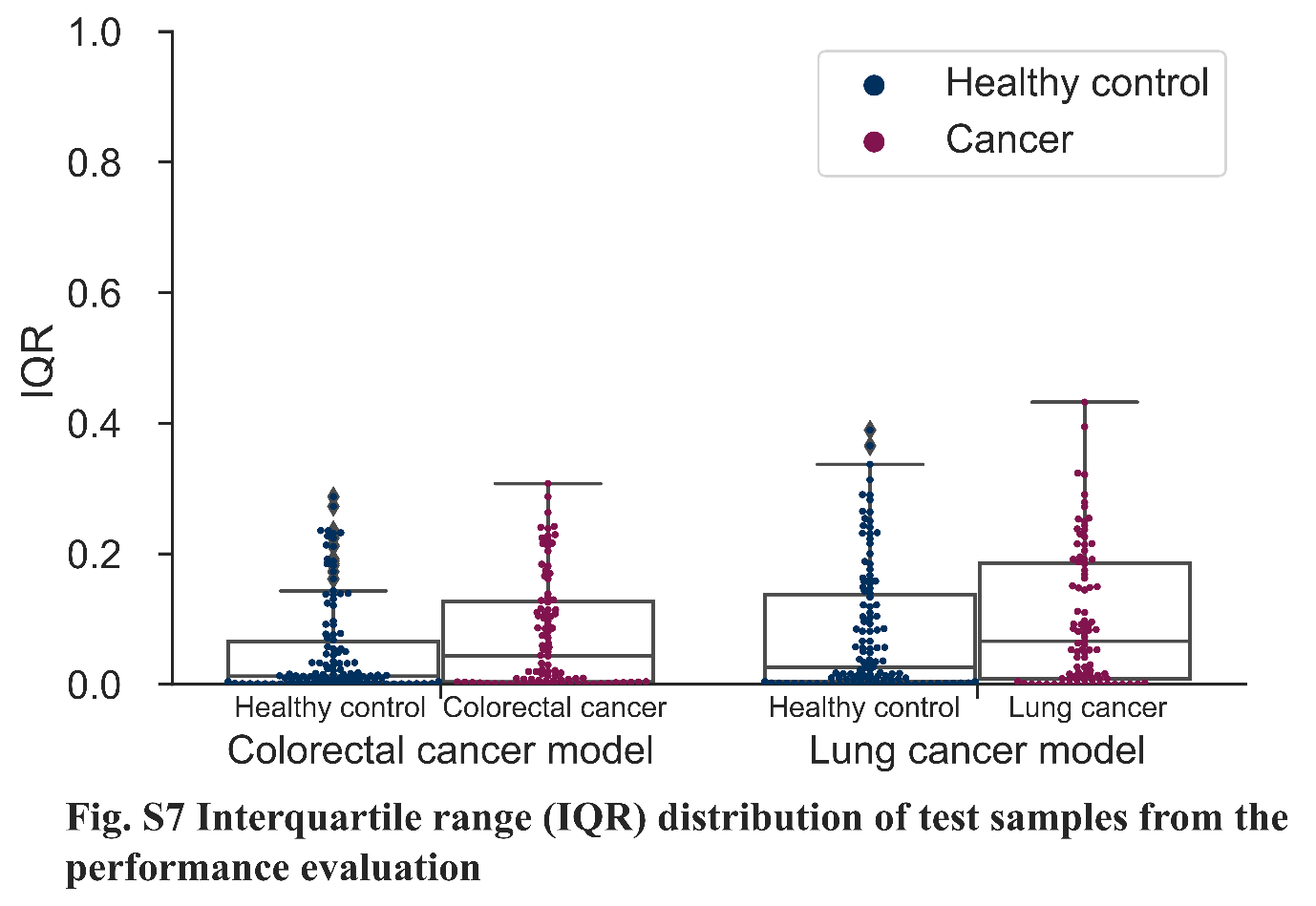


Through the 20 repetitions of 25 trainset combinations, a total of 500 cycles of cross-validation were performed, and each test sample was tested 100 times. A lower IQR entailed the acquisition of a more stable result

**
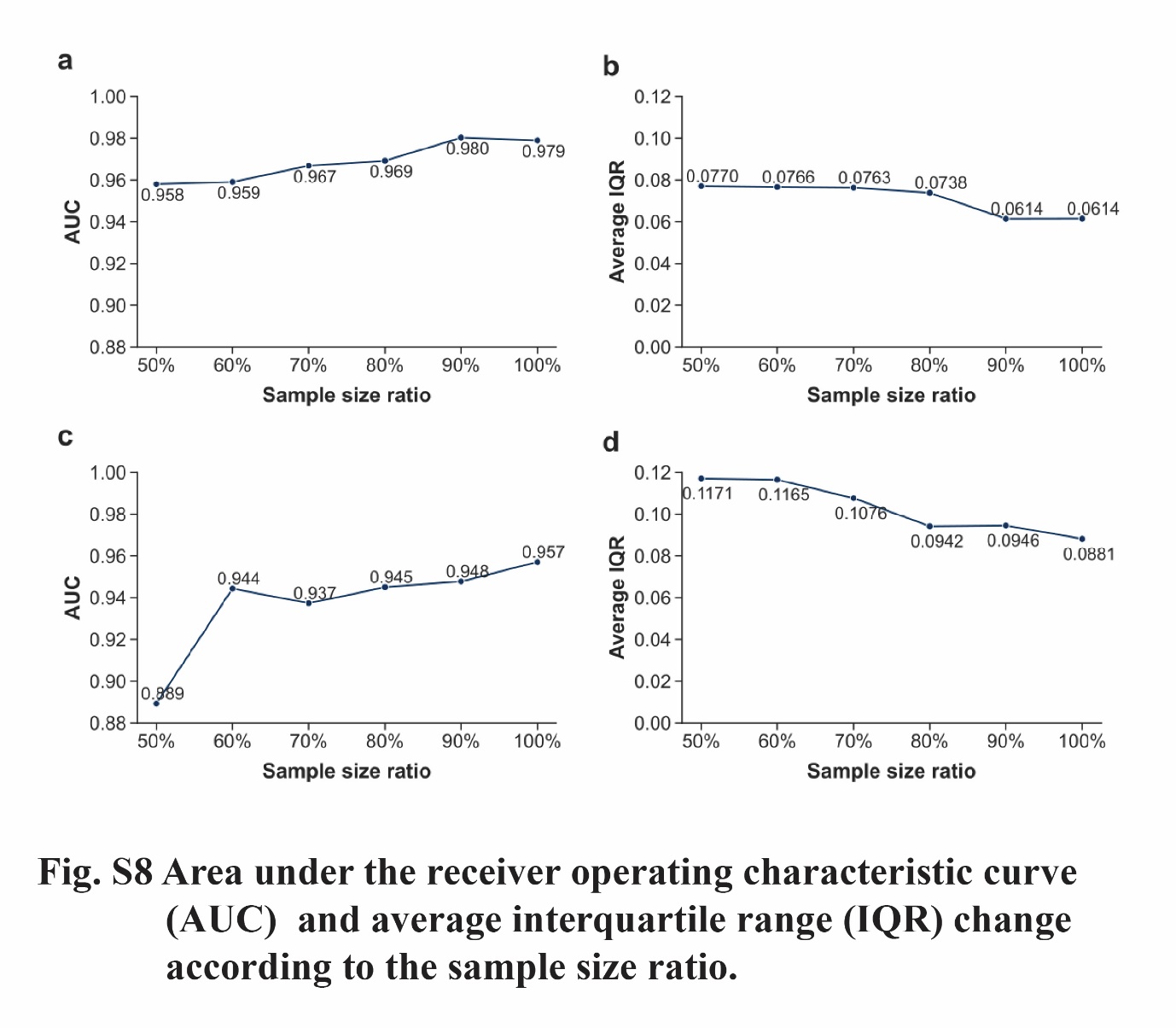
**

**a** and **b** Changes in the AUC and average IQR of the colorectal cancer model. **c** and **d** Changes in the AUC and average IQR of the lung cancer model

**
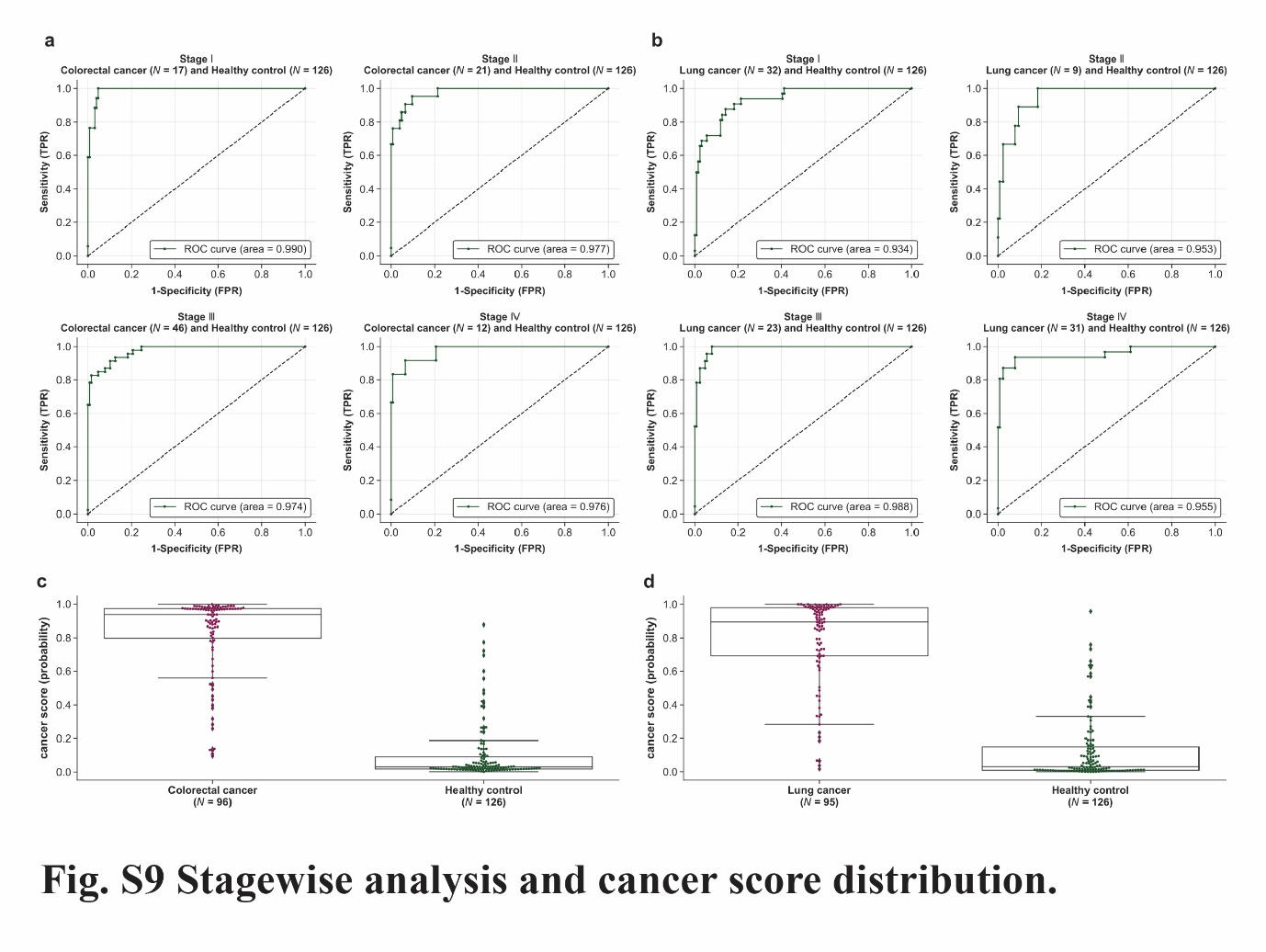
**

**a** and **b** Stagewise receiver operating characteristic (ROC) of colorectal and lung cancers (stages I–IV). **c** and **d** Score distribution between cancer and healthy control samples

**
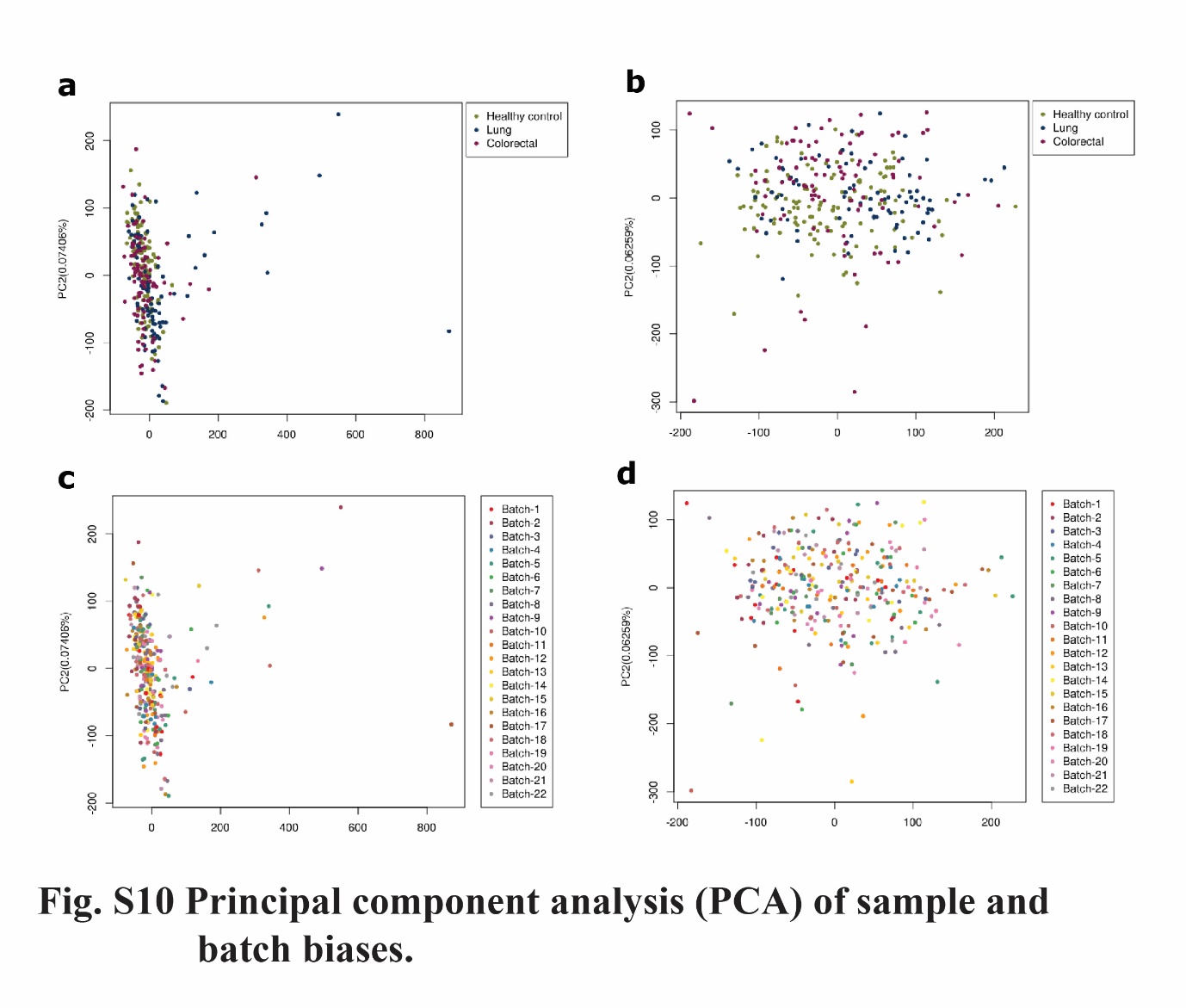
**

Confirmation of unbiased dataset by PCA analysis. **a** and **b** Three sample groups (normal, colorectal, and lung) including and excluding nine outliers. **c** and **d** Individual sequencing batches including and excluding the nine outliers


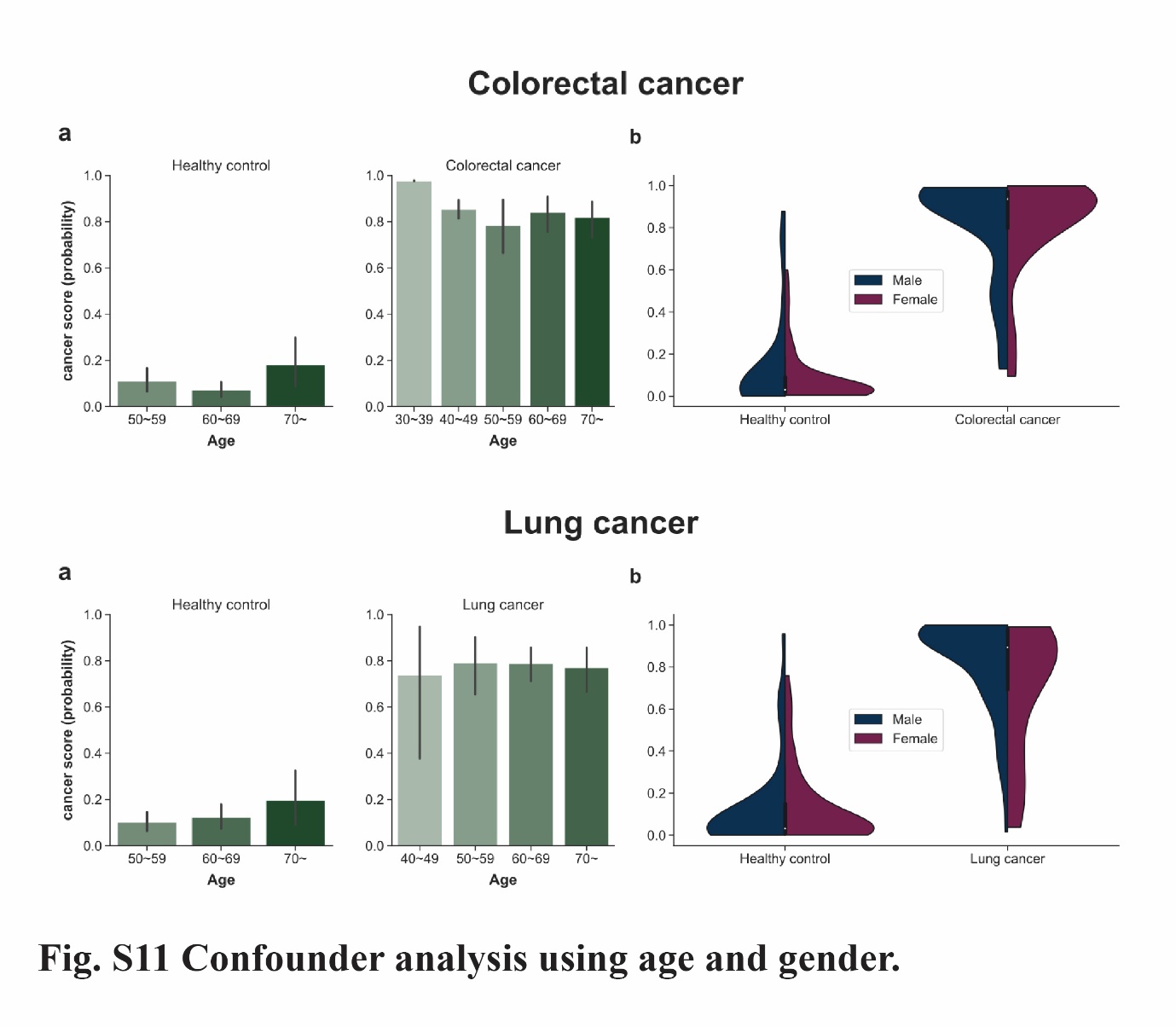


**a** Binned-age histogram with cancer score. **b** Cancer score distribution between men and women


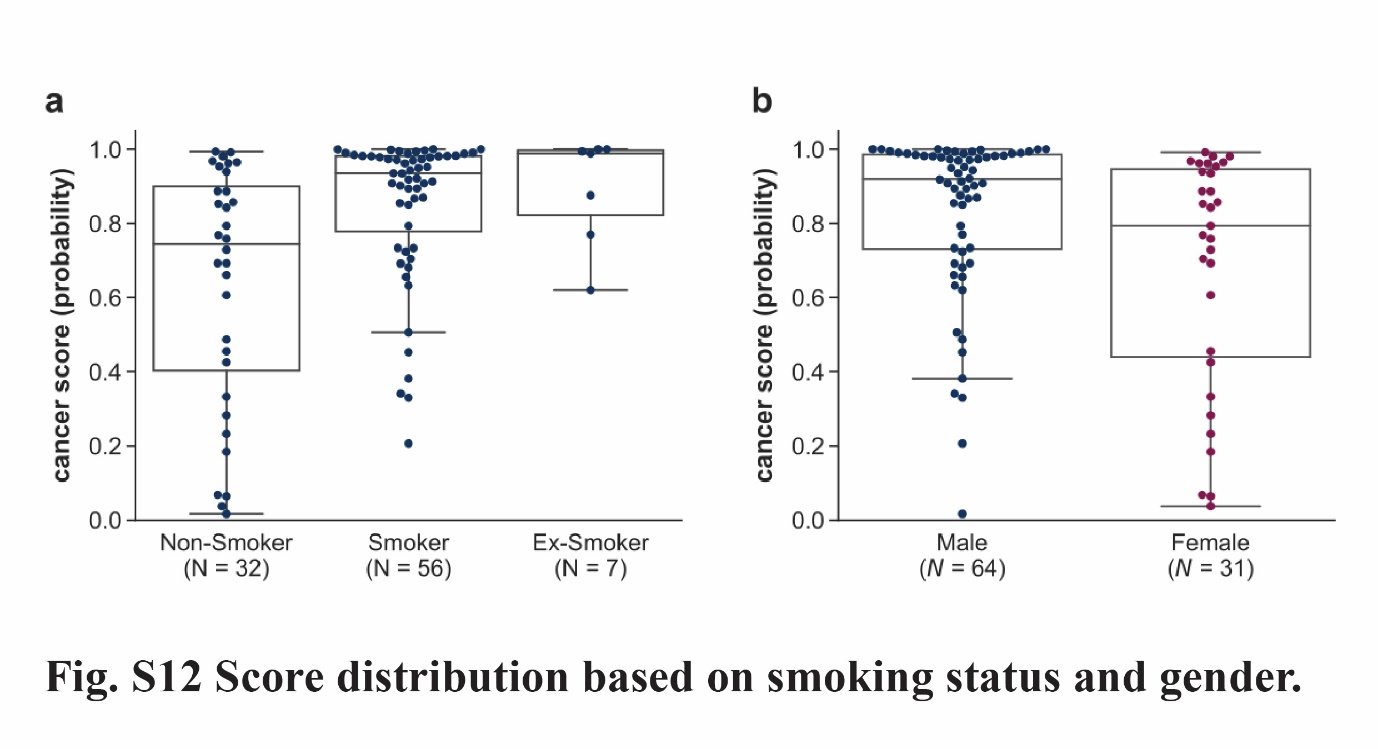


**a** The average cancer score for nonsmokers was 0.63, whereas for smokers and ex-smokers, the average cancer scores were 0.85 and 0.89, respectively. **b** The average cancer scores were 0.83 and 0.68 for males and females, respectively

**Method: Performance assessment and hyperparameter tuning**

Model performance evaluation and hyperparameter tuning were performed using Python version 3.7 and TensorFlow [1] 2.4 graphics processing unit (GPU) docker in the Linux Ubuntu 20.0 environment, and K-fold cross-validation was performed with scikit-learn 1.0.1 [2].

For hyperparameter tuning, fivefold cross-validation was repeated 25 times, for a total of 125 cycles. During each validation cycle, randomly stratified 80% of the total samples were used for hyperparameter tuning and model training. Finally, the set of hyperparameters that appeared most frequently in the total 125 cycles was determined as the best parameter set (Supplementary Fig, 2a). The number of nodes in the hidden layer was different between the colorectal and lung cancer models, with 32 and 64 nodes, respectively. Moreover, two hidden layers and an epoch of 120 were common in both models by applying the Adam optimizer and rectified linear unit activation function (Supplementary Fig. 3).

In this paper, the repeated K-fold cross-validation was used to measure the model’s performance. The shuffling and classification of healthy control and the cancer sample sets into five folds each allowed the creation of a total of 25 test pairs. At this time, stratification was performed on healthy control and cancer samples so that each sample group was selected at the same ratio. By performing K iterations (K = 25) and repeating this process 20 times, one sample was being tested 100 times, and the final performance value was calculated as the average value of each test score (Supplementary Fig. 2b). Through the distribution of the score of one test sample, we can measure the distribution of the interquartile range (IQR) or the standard deviation to determine the robustness and consistency of the results.

**References**

1. Abadi M, Barham P, Chen J, Chen Z, Davis A, Dean J, et al. TensorFlow: A system for large-scale machine learning. 2016. https://doi.org/10.48550/ARXIV.1605.08695.

2. Pedregosa F, Varoquaux G, Gramfort A, Michel V, Thirion B, Grisel O, et al. Scikit-learn: Machine Learning in Python. 2018.
